# Impact of hydrocolloid and foaming agent on the physicochemical, microstructural and bioactive characteristics of foam‐mat freeze‐dried tapai (fermented black glutinous rice) powder

**DOI:** 10.1002/fsn3.3098

**Published:** 2022-10-07

**Authors:** Nur Fauziyah, Idolo Ifie, Osman Syarief, Sandi Darniadi

**Affiliations:** ^1^ Department of Nutrition Politeknik Kesehatan Bandung Indonesia; ^2^ School of Food Science and Nutrition University of Leeds Leeds United Kingdom; ^3^ Department of Food Science and Technology Delta State University Abraka Nigeria; ^4^ Research Center for Appropriate Technology National Research and Innovation Agency (BRIN) Subang Indonesia; ^5^ Department of Food Technology University of Pasundan Bandung Indonesia

**Keywords:** black glutinous rice, foaming agent, foam‐mat freeze‐drying, hydrocolloid, powder

## Abstract

The aim of this study was to obtain fermented black glutinous rice (FBGR) powder using the foam‐mat freeze‐drying method and to evaluate the physicochemical, microstructural, and bioactive characteristics of FBGR powder. To obtain FBGR foams, maltodextrin (MD; 0%–40% w/w) and whey protein isolate (WPI; 7.5% w/w) were used. The results showed that MD concentration had a significant effect (*p* ≤ .05) on powder recovery and bulk density which increased from 68.9% to 80.5% and 0.46% to 0.63 g/ml, respectively. The lowest moisture content (2.17%) and water activity (0.48) were found in powders produced with 20% MD. The solubility of FBGR powders ranged from 74% to 75% and increasing MD concentration gave higher lightness (L*) and yellowness (b*) readings in color properties of powders. Although the addition of the carrier agent caused reductions in total phenolic content and anthocyanins, the retention of these bioactive compounds rose with increasing MD content from 67% to 76% and 78% to 84%, respectively. FBGR produced with 40% MD had the most superior physical and technological properties, and foam‐mat freeze‐drying is a promising technology for retaining the bioactive compounds in FBGR.

## Graphical abstract

Foam‐mat freeze‐drying, using different concentrations of maltodextrin (MD) and whey protein isolate (WPI), represents a promising technology for retaining the bioactive compounds in fermented black glutinous rice (FBGR) powder and have the most superior physical and technological properties.

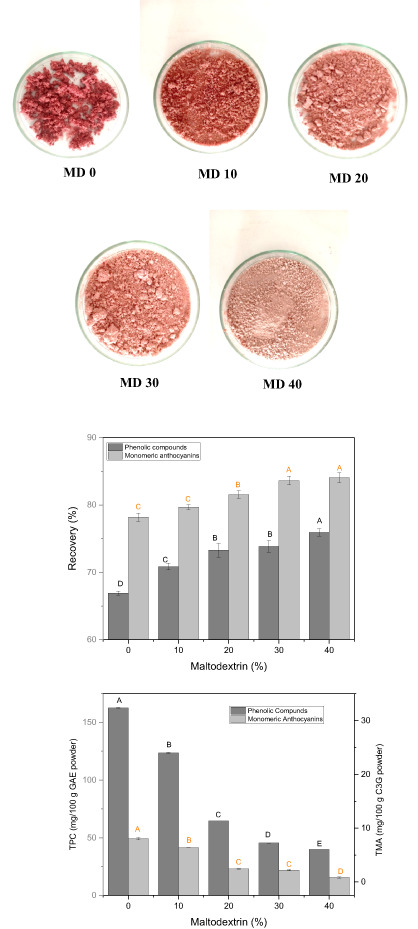

## 1 INTRODUCTION

Black rice (*Oryza sativa* L.) is one of the main traditional rice crops in many countries of Southeast Asia (Kanha et al., 2020). The phytochemical constituents of pigmented rice are flavonoids, phenolic compounds, tannins, sterols, and essential oils (Chaiyasut et al., 2017). It also contains anthocyanin pigments, with the dominant being cyanidin‐3‐glucoside (92.5%) and peonidin‐3‐glucoside (7.5%). Cyanidin‐3‐glucoside, in particular, has been shown to have strong antioxidant activity and has been reported to have beneficial effects on human health, such as oxidative stress and anticancer effects (Castañeda‐Ovando et al., 2009).

One of the products obtained from black rice, primarily the glutinous type, is fermented black glutinous rice, which is ethnic to Indonesia (Fauziyah, Abdillah, et al., 2020; Fauziyah, Afiani, et al., 2020; Syarief et al., 2020). FBGR is sweet in taste and commonly served as dessert on special occasions. FBGR is produced by soaking the black glutinous rice for 12 h and then steaming it for 2 h at 98°C. The sample is chilled, combined with yeast cake starter *Saccharomyces cerevisiae* (0.27% w/w of the sample), and fermented for 3 days in a plastic bag at room temperature (25–27°C; Sarida et al., 2016). FBGR product contains 25.7 mg of anthocyanin, 5.9 g of fiber in 100 g, and has total sugar content reaching up to 18.39% (Fauziyah, Abdillah, et al., 2020; Fauziyah, Afiani, et al., 2020; Syarief et al., 2020). The consumption of FBGR anthocyanin has been reported to be effective against cardiovascular disease, diabetes mellitus, and cancer by acting as an antioxidant, anti‐inflammatory, and anticancer agent (Fauziyah, Abdillah, et al., 2020; Fauziyah, Afiani, et al., 2020).

The utilization of the FBGR needs to be adopted widely and not limited to its country of manufacture. A major drawback to its utilization is the limited shelf‐life which is about 7–10 days under refrigerated conditions (Trinovani et al., 2020). Therefore, improving the processing of FBGR using appropriate processing methods can lead to the design of ready‐to‐eat foods with longer shelf lives and superior bioactive compounds.

Among existing processing methods, spray‐drying and freeze‐drying are recommended for food powder production (Turan et al., 2016). Spray‐drying is a conventional method extensively employed to produce food powder and food ingredients in many industries. Spray‐drying has various advantages; it is a continuous process, making it an economically viable technology with low residence durations (Turan et al., 2015). Conversely, spray‐drying uses relatively high drying temperatures (>100°C) which could increase the degradation of anthocyanins (Fracassetti et al., 2013). With its low operating temperatures, freeze‐drying may be an excellent method for producing powder from high‐sugar foods (Seerangurayar et al., 2017b). Additionally, it provides superior rehydration characteristics for dry items. However, the primary disadvantage of this procedure is the duration of time required for drying. To shorten the freeze‐drying time, foaming is used prior to the dying process (Izquierdo‐López et al., 2017).

The process of foam‐mat freeze‐drying commonly used in the food business entails producing a stiff foam by whipping in foaming agents/foam stabilizers to a solution (Macedo et al., 2021). Maltodextrin (MD) and whey protein isolate (WPI) are often used as a carrier and foaming agent in the drying process because they form a film when dry, which helps to remove the sticky surface of sugar and protein solutions (Celli et al., 2016; Fang et al., 2013). In addition, these carrier agents act as effective protection of bioactive compounds during the drying process (Norkaew et al., 2019). In spite of their advantages, the amount of foaming agent is a determining factor in the structure of foams (Jakubczyk & Kamińska‐Dwórznicka, 2021). Therefore, investigating the concentrations of foam stabilizers and foaming agents is critical to producing the most desirable foam characteristics for each food type.

To the best of our knowledge, data on the application of foam‐mat freeze‐drying techniques for producing FBGR powder are scarce. Thus, this work aimed at manufacturing a foam‐mat freeze‐dried FBGR powder with the addition of MD and WPI, and exploring the physicochemical and techno‐functional characteristics of the final product.

## 2 MATERIALS AND METHODS

### 2.1 Materials

Fermented black glutinous rice (FBGR) was purchased from the local market in Bandung, Western Java, Indonesia. The FBGR product was then stored in the fridge at 4–8°C before processing for juice preparation. Maltodextrin/MD (Sigma Aldrich; 16.5–19.5 dextrose equivalent) and whey protein isolate (WPI; Fonterra) were utilized as foam stabilizer and foaming agent, respectively.

### 2.2 Foam‐mat Freeze‐drying of FBGR

FBGR extract preparation was done by mixing (2:1 w/w) FBGR paste and water using a food processor (Russell Hobbs, UK), with the final concentration of soluble solids reaching 14 ^0^brix. The foaming process of the juice, which aimed to obtain the dried powder, was conducted by the addition of MD (0%, 10%, 20%, 30% and 40% w/w) and WPI of 7.5% w/w (Darniadi et al., 2020;Ozcelik, Ambros, et al., 2019; Ozcelik, Heigl, et al., 2019). The MD and WPI were added to the juice and then whipped by a hand mixer (Russell Hobbs) at high velocity for 10 min. The foam was then transferred into a stainless‐steel tray (2–3 mm thick).

The foam‐mat freeze‐drying was done using the method described by Darniadi et al. (2020). The foamed juice was placed inside a freezer (−40°C) for 24 h. The frozen samples were then transferred into the freeze dryer (Buchi Lyovapor L‐200) operated at condenser temperature −55°C and vacuum pressure of 0.04 mbar for a 48 h duration. The freeze‐dried samples were then crushed for 1 min employing a Kenwood CH 180A mini chopper food processor (Kenwood). The FBGR powders were weighed and transferred into air‐tight vessels and kept in a refrigerator at 5^°^C for further analysis.

### 2.3 Texture of dried foams

A Brookfield Pro CT3 texture analyzer (AMETEK Brookfield) having a 50‐kg load cell and the Kramer shear cell was used to examine the texture of dry foams. The Kramer shear cell (KSC) with five blades was selected due to its ability to analyze multiparticle products. During the analysis, the dried samples were compressed, sheared, and extruded in a mixture of compression, shearing, and extrusion (Ozcelik, Ambros, et al., 2019; Ozcelik, Heigl, et al., 2019). The weight of the sample tested was 10 g under the same condition in triplicate. Force versus time data were gathered using the test equipment's proprietary software Texture Pro CT (AMETEK Brookfield). The greatest peak force is given in terms of the sample's “hardness” (*N*).

### 2.4 Powder recovery

The powder recovery was calculated as the ratio of the mass of solid powder produced after the freeze‐drying to the mass of beginning substances dried, which includes both MD and WPI (Shi et al., 2013).

(1) $$\text{Powder recovery}\;\left(\%\right)=\frac{\text{Solids in powder}}{\text{Total solids in foam}}\times 100\;$$

### 2.5 Bulk density

The bulk densities of the powders were calculated by gently loading 1 g of FBGR powder into an empty 10 ml graduated cylinder and holding the cylinder at 2000 rpm for 1 min on a Fischer Scientific FB 15012 top mix vibrator (Fischer Scientific). The bulk density of the product was determined by dividing its mass by the volume filled by the cylinder (Yüksel, 2021).

(2) $$\text{Bulk density}\;\left(\rho B\right)=\frac{\text{Mass of powder}\;\left(g\right)}{\text{Volume of powder}\;\left(\mathrm{ml}\right)}$$

### 2.6 Solubility

Solubility was determined as described by Rin‐ut and Rattanapitigorn (2020) with slight modification. Briefly, 1 g of powder samples were gradually dissolved in 100 ml of distilled water (30^°^C) and stirred with the aid of a Stuart CB‐162 magnetic stirrer (Bibby Scientific Ltd) operated at 400 rpm for 5 min. The dispersions were placed inside falcon tubes (50 ml) and centrifuged using a Universal 320 centrifuge (Sartorius) set at 3000 rpm for 10 min. Twenty‐five milliliter of the resulting supernatant was then placed inside a preweighed Petri dish and oven‐dried at 105^°^C for 5 h. The water solubility was calculated using the formula:

(3) $$\text{Water solubility}\;\left(\%\right)=\frac{\left(\mathrm{WA}-\mathrm{WB}\right)}{\mathrm{WX}}\times 100$$

where WA is weight (g) of supernatant before drying, WB is weight (g) of supernatant after drying, and WX is weight (g) of the sample.

### 2.7 Rehydration time

The method of Islam et al. (2016) was employed for the determination of rehydration of FBGR samples. Briefly, 0.5 g of sample powder was weighed and transferred to a 100 ml glass beaker. Thereafter, 50 ml of distilled water at 30°C was added to the sample and the mixture was agitated using a Stuart CB‐162 magnetic stirrer (Bibby Scientific Ltd) at 900 rpm. The time duration for all particulate matter to be invisible to the naked eye was then recorded.

### 2.8 Color (L*a*b* values)

The color parameters of fermented black glutinous rice (dried powder) were recorded using a Minolta CR‐200 (MINOLTA Camera Co., LTD) brand Chroma meter with illuminant D65 and 10^∘^ observer angle. The color indices evaluated were L* (lightness), a* (redness), and b* (yellowness) values (Turan et al., 2016).

### 2.9 Microstructural structure

A field‐emission scanning electron microscope (FE‐SEM; Zeiss Sigma, Carl Zeiss Microscopy) was employed to determine the microstructure of the dried samples. After coating the samples with 10 nm of gold–palladium, SEM images were recorded in high vacuum mode using a secondary electron with an accelerating voltage of 5 kV, and the magnification used was 250× (Ozcelik, Ambros, et al., 2019; Ozcelik, Heigl, et al., 2019).

### 2.10 Moisture content and water activity

To analyze the moisture content in the samples, an MB‐120 Halogen Moisture Analyser set at 105°C was employed for the analysis (Darniadi et al., 2020). One gram of powder sample was measured and put inside the sample pan, after which the moisture analyzer's lid was lowered until it was tightly closed. For each sample, the drying time was between 2 and 3 min. For water activity determination, approximately 0.5 g of powders were measured and the analysis was conducted using a Hygrolab C1 water activity meter (Fang & Bhandari, 2012).

### 2.11 Proximate analysis

The crude fat was determined by continuous extraction in a Soxhlet apparatus for 18 h with hexane as the solvent, the ash by incineration at 550°C, the crude fiber by sequential hot digestion of the defatted samples with dilute acid and alkaline solutions, and the total carbohydrate by difference (AOAC, 2005). The crude protein content was determined using Kjeldahl's method (Atasie et al., 2009), and the nitrogen content was determined using a spectrophotometric method given by Atasie et al. (2009). Each analysis was carried out in triplicate. Energy was calculated as follow (Dewi et al., 2020): Energy (Kcal) = [(carbohydrate + protein) × 4] + [Fat × 9].

### 2.12 Dietary fiber content and total sugars

The established enzymatic–gravimetric procedure (AOAC 991.43) was used for the measurement of both insoluble (IDF) and soluble dietary fiber (SDF) contents (AOAC, 2005). Total sugar analysis was determined by the method of AOAC (2005).

### 2.13 Total phenolic content, total monomeric anthocyanins, and recovery

Analysis of total phenolic content was performed for both FBGR and their reconstituted powders. The total phenolic content (TPC) was determined using Folin–Ciocalteu's method described by Ifie et al. (2016). In addition, total monomeric anthocyanin (TMA) in FBGR powders was examined based on the pH differential method (Darniadi et al., 2019). The test portions were diluted with pH 1.0 and pH 4.5 buffers and the absorbance at 520 and 700 nm was measured with a 6715 UV/VIS spectrophotometer (Jenway). The diluted test portions were red versus a blank cell filled with distilled water using a 10 ‐mm‐route‐length glass cuvette. The concentration of anthocyanin pigment was then determined and expressed as cyanidin‐3‐glucoside equivalents, as shown below.

(4) $$\text{Total Monomeric Anthocyanins}=\frac{\left(A\times \mathrm{MW}\times \mathrm{DF}\times 1000\right)}{\epsilon \times l}$$

where A = (absorbance at 520 nm − absorbance at 700 nm) at pH 1.0 − (absorbance at 520 nm − absorbance at 700 nm) at pH 4.5; MW (molecular weight) = 449.2 g/mol for cyanidin‐3‐glucoside (Cyn3Gl); DF = dilution factor; l = path length in cm; *Ɛ* = 26,900 molar extinction coefficient, in L/mol × cm, for Cyn3Gl; and 1000 = factor for conversion from g to mg.

The method of Fang and Bhandari (2011) was employed to calculate TPC and TMA recovery using the following formula (expressed as dried matter):

(5) $$\mathrm{TPC}\;\text{recovery}\;\left(\%\right)=100\times \frac{\mathrm{TPC}\;\text{in powder}}{\mathrm{TPC}\;\text{in juice}}$$

(6) $$\mathrm{TMA}\;\text{recovery}\;\left(\%\right)=100\times \frac{\mathrm{TMA}\;\text{in powder}}{\mathrm{TMA}\;\text{in juice}}$$

### 2.14 In vitro antioxidant activity

The DPPH free radical scavenging assay was conducted using the protocol reported by Molyneux (2004). Standard solutions or DMSO (control) were made and added to a 70 lmol/L DPPH methanolic solution; after vigorous shaking, the mixtures were allowed to stand at room temperature for 30 min in the dark before reading the absorbance at 517 nm. The radial scavenging capacity was expressed in terms of a percentage effect (*E*%) based on the following equation:

(7) $$\text{Percentage effect}\;\left(E\%\right)=\frac{\mathrm{Abs}\;\text{control}-\mathrm{Abs}\;\text{sample}\;}{\mathrm{Abs}\;\text{sample}}\times 100$$

Various sample concentrations were utilized to generate antiradical curves for the purpose of estimating the EC50 values. Antiradical curves were produced using the x‐axis to indicate concentration and the y‐axis to indicate relative scavenging capacity.

### 2.15 Statistical analysis

The processing treatments were triplicated and their mean values determined. Analysis of variance was done to ascertain if significant differences existed between means. Multiple comparisons were performed using the Tukey test, and the significance level was established at *p* ≤ .05. The statistical package used for the analysis was Minitab 19.0.

## 3 RESULTS AND DISCUSSION

### 3.1 Texture of dried foams

**FIGURE 1:**
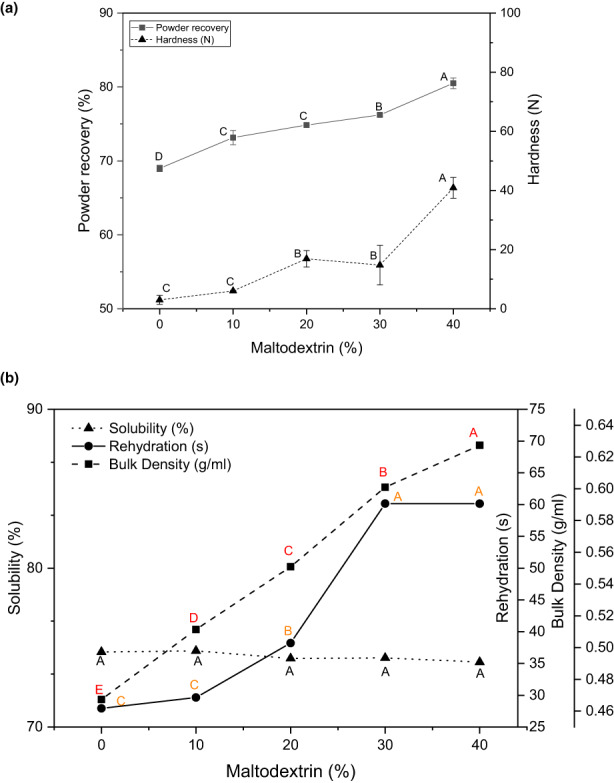
(a) Hardness of FBGR dried foams and powder recovery of foam‐mat freeze‐dried FBGR powder. (b) Bulk density, rehydration, and solubility of foam‐mat freeze‐dried FBGR powder

After freeze‐drying, the dried layer was subjected to textural property analysis using a texture analyzer. Figure 1 presents the hardness level of FBGR dried foam, which was influenced by the incorporation of MD (0%–40%). The hardness of FBGR foams observed ranged from 2.9 to 40.8 N, where higher MD content intensified the hardness of dried foams. The data observed are in line with the study of Ozcelik, Ambros, et al. (2019); Ozcelik, Heigl, et al. (2019), who found that adding MD into raspberry juice increased the hardness level. Similarly, the bulk density of MD 40 concentration was higher than all other samples with lower MD concentrations (Figure 1), and the hardest dried foam was made with 40% (w/w) MD. This correlation between density and hardness suggests that, in addition to the effect on density, the bubble size and distribution significantly impacted sample hardness (Ozcelik, Ambros, et al., 2019; Ozcelik, Heigl, et al., 2019). A narrow, homogeneous pore structure necessitates more power to shear and compresses the foam, resulting in more rigid foams.

### 3.2 Powder recovery

Powder recovery is an essential indicator that shows the efficiency of the drying process. Figure 1 shows powder recovery values ranging from 68.9 to 80.5% and that the recoveries increased with higher MD contents. In a previous study, Darniadi et al. (2019) reported improved recovery of freeze‐dried blueberry powder with MD and WPI and a yield of approximately 72%–79%. Other researchers have also reported higher feed solid recovery with increased MD content (Chasekioglou et al., 2017; Shrestha et al., 2007). The increased powder recovery can be attributed to the film‐forming properties of MD and WPI, which, upon drying, are able to overcome the surface stickiness of sugar/protein solutions (Fang et al., 2013; Shi et al., 2013).

### 3.3 Bulk density

The bulk density of powdered foods is a pointer to the ease of reconstitution, packaging, transportation, and storage (Fitzpatrick, 2013). In addition, powder attributes such as flowability are enhanced by higher bulk densities (Fitzpatrick, 2013; Gallo et al., 2011). In this study, following the addition of MD, the bulk density of foam‐mat freeze‐dried FBGR increased from 0.46 to 0.63 g/ml (Figure 1b). Seerangurayar et al. (2017b) reported values of 0.60–0.68 g/ml after using 40%–50% MD in the production of foam‐mat freeze‐dried date powders. Michalska & Lech (2018) also showed that increasing MD concentration (15%–35%) led to higher bulk density values (0.53–0.61) in apple juice powders. According to the authors, this may be due to less crystallization at higher MD concentrations, resulting in greater bulk density. Similar results were observed in the preparation of avocado oil powder, where bulk density increased as MD ratio increased (Bae & Lee, 2008). In dried beetroot powder manufactured with and without MD, the samples devoid of MD exhibited the lowest bulk density (Ng & Sulaiman, 2018).

### 3.4 Solubility

A vital property of food powders is their solubility because powders with poor solubility provoke challenges during processing resulting in economic losses (Sharma et al., 2012). For food powders to be useful and functional, they must have high solubility in the aqueous phase. FBGR powder samples, regardless of carrier agent concentration, exhibited solubility in the range 74%–75% (Figure 1b). Based on the powder solubility data, the results in this study are similar to those in previous studies on Mao powders (89.29%–96.87%), black glutinous rice bran powders (76.23%–91.79%), and pomegranate juice powders (92%–95%; Laokuldilok & Kanha, 2015; Suravanichnirachorn et al., 2018; Yousefi et al., 2011). A more recent study showed that solubility for Andean blueberry juice freeze‐dried powders containing MD concentrations of 20%–50% (w/w) varied from 92% to 94% (Estupinan‐Amaya et al., 2020). The water solubility of freeze‐dried powders is reported to depend on the morphology, particle size, and interparticle voids of powders, as well as the characteristics of juices and carriers (López‐Córdoba & Goyanes, 2017).

### 3.5 Rehydration

Powdered ingredients are often dissolved before use, so it is important that they rehydrate easily in aqueous media (Felix da Silva et al., 2017). Figure 1b showed that rehydration time rose from 20 to 60 s with increasing MD concentration. Red beetroot powder produced by foam‐mat freeze‐drying at varying MD concentrations (0%–40%) rehydrated within 28–40 s, with 40% MD taking the longest time to rehydrate (Hajiaghaei & Sharifi, 2022). This can be attributed to MD belonging to the modified starch family, which has a longer dissolution time than most hydrophilic components in the plant extracts (Caliskan & Nur Dirim, 2013). Thus, it is expected that FBGR with higher MD concentration will have longer rehydration times (Nunes et al., 2015).

### 3.6 Color (L*a*b* values)

**FIGURE 2:**
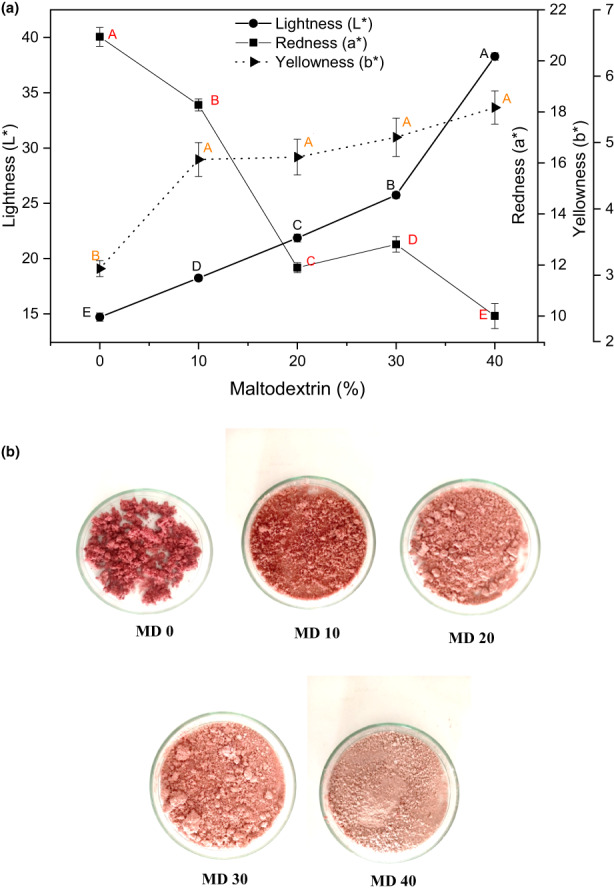
(a) Color of foam‐mat freeze‐dried FBGR powder. (b) Foam‐mat freeze‐dried FBGR powder produced with WPI 7.5% and various MD concentrations

A vital quality parameter influencing consumers' choice of dried food is its color. To make food products more appealing to consumers, the color of the supplemented products should ideally be close to their natural state. Based on the data on color parameters, redness (a*) decreased with increasing MD concentration, while lightness (L*) and yellowness (b*) of samples increased (Figure 2a,b). Similar trends were observed in powdered sumac extract, mountain tea, purple sweet potato, and instant soluble sage after the addition of MD in the drying process (Ahmed et al., 2010; Caliskan & Nur Dirim, 2013; Şahin Nadeem et al., 2011; Şahin‐Nadeem et al., 2013). The color of MD is white, consequently increasing the amount of MD increases the L* value of the powders. Similarly, decreasing redness (lower a* values) in powder after drying following the addition of MD has also been encountered in other studies (Jaya et al., 2006).

### 3.7 Microstructural structure

A particle's shape can be used as a filter before its size is determined to define its behavior. The SEM images of the processed FBGR powders are shown in Figure 4. The particles of the control powders were irregular in shape and had a rough shriveled surface. By contrast, FBGR powder obtained with MD 10%–40% was irregular in shape, hairy, smooth, and agglomerated on the surface. Furthermore, the powders produced with MD varied in their microstructure. This variation might be attributed to the molecular assembly of carrier agents (Fazaeli et al., 2012; Seerangurayar et al., 2017a). According to Du et al. (2014), freeze‐dried persimmon powder exclusive of carrier agents possessed irregular flakes. This agrees with the findings of the current research.

### 3.8 Moisture content and water activity

The moisture content in the dry extract is an essential property for determining their storage stability, handling, and flowability since it affects the glass transition and crystallization behavior (Jokić et al., 2020). In addition, the drying efficiency is a factor influenced by the moisture content of a sample (Ferrari et al., 2012). According to Table 1, moisture content decreased from 9.2% to 2.4% with increasing MD content. The reduction in the powders' hygroscopicity with increasing MD content may have influenced the moisture content of the samples. Additionally, the reduced moisture content may also be due to the increased carbohydrate concentration, which increases the sample solids and decreases the amount of moisture that can be evaporated (Ekpong et al., 2016; Tchabo et al., 2018). Our findings are consistent with published data on spray‐dried tamarind, persimmon, and gac fruit pulp powders (Bhusari et al., 2014; Du et al., 2014; Kha et al., 2010). Furthermore, the moisture content observed in this study falls within values reported for foam‐mat dried muskmelon (2.3%–2.7%), shrimp (1%–4%), and yacon (3.5%–6.6%) powders (Asokapandian et al., 2016; Azizpour et al., 2016; Franco et al., 2016).

**TABLE 1:** Proximate, total sugars, and dietary fiber of foam‐mat freeze‐dried FBGR powder

Properties	Maltodextrin (%)				
	0	10	20	30	40
Moisture (%)	9.24 ± 0.05^a^	6.10 ± 0.05^b^	2.17 ± 0^d^	2.18 ± 0.01^d^	2.44 ± 0.04^c^
Water activity	0.55^a^	0.49^c^	0.48^c^	0.48^c^	0.52^b^
Carbohydrate (%)	79.52 ± 016^e^	86.74 ± 0.08^d^	92.48 ± 0.23^c^	92.91 ± 0.21^b^	93.39 ± 0.04^a^
Protein (%)	9.13 ± 0.08^a^	5.98 ± 0.18^b^	4.28 ± 0.23^c^	3.99 ± 0.17^c^	3.39 ± 0.02^d^
Fat (%)	0.54 ± 0.02^a^	0.31 ± 0.01^b^	0.28 ± 0.06^bc^	0.27 ± 0.00^bc^	0.21 ± 0.05^c^
Ash (%)	1.57 ± 0.03^a^	0.87 ± 0.04^b^	0.80 ± 0.00^c^	0.65 ± 0.01^d^	0.57 ± 0.01^e^
Energy (Kcal)	359.43 ± 0.30^d^	373.63 ± 0.33^c^	389.47 ± 0.00^b^	390.06 ± 0.34^ab^	389.01 ± 0.36^a^
Total sugars (%)	54.57 ± 0.26^a^	46.85 ± 0.28^b^	39.78 ± 0.35^b^	33.69 ± 0.16^d^	25.58 ± 0.43^e^
Dietary fiber (%)	2.78 ± 0.06^e^	3.10 ± 0.02^d^	3.44 ± 0.03^c^	3.60 ± 0.07^b^	3.74 ± 0.07^a^

*Note*: Different superscript letters a–e in row indicate significant difference (*p* < .05).

An important factor in the stability of food powders is water activity (a_w_), which influences both chemical and microbial stability. In this study, the water activity decreased from 0.55 to 0.46 as MD concentration increased from 0 to 40% (Table 1), indicating samples' stability and possible inhibition of microbial growth (Shi et al., 2013). The same findings were also reported in a previous study of spray‐dried watermelon, where higher carrier concentration (MD) resulted in lower aw values (Quek et al., 2007). The water activity observed in our study (Figure 2b) was found to be lower or similar to values obtained for probiotic orange powders (0.34–0.42), powders of spray‐dried watermelon (0.20–0.29), and microencapsulated Andes berry extracts (0.199–0.422), respectively (Barbosa et al., 2015; Quek et al., 2007; Villacrez et al., 2014).

### 3.9 Proximate analysis, total sugars, and dietary fiber content

The proximate composition of FBGR powder samples is presented in Table 1. The data showed an increase in both carbohydrate and energy content with increasing MD. This trend can be justified since MD is a polysaccharide that invariably contributes to the energy content of the product. In contrast, FBGR powder protein, ash, fat, and fat content decreased as observed in Grabowski et al.'s (2008) study using MD to produce sweet potato powder. The data on total sugars in samples is presented in Table 1. Total sugars of foam‐mat freeze‐dried FBGR were in a range 25.58%–54.57%. Total sugars reduced with increasing MD concentration in samples. In the presence of MD, the fiber content increased from 2.78 to 3.74 (Table 1). The increase in dietary fiber can be credited to the MD that provides a source of dietary soluble fiber.

### 3.10 Total phenolic, monomeric anthocyanins, antioxidant activity, and recovery

**FIGURE 3:**
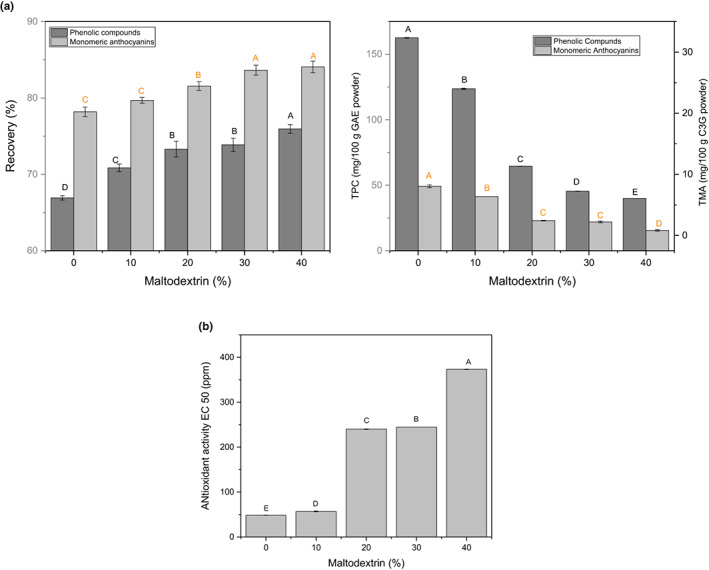
(a) TPC, TMA, and recovery of foam‐mat freeze‐dried FBGR powder. (b) Antioxidant activity of foam‐mat freeze‐dried FBGR powder

Polyphenols represent a class of plant secondary metabolites attributed to biological effects such as antioxidant, antiviral, antibacterial, and anticancer. The data on total phenolic and monomeric anthocyanin contents in the sample, as reflected in Figure 3a, showed that as concentrations of MD increased, both TPC and monomeric anthocyanin contents decreased considerably ranging from 24% to 75% and 16% to 69%, respectively. This might be connected to the introduction of the carrier agent (MD), which could dilute and temper the product's phenolic content, resulting in lower TPC and monomeric anthocyanins (Hajiaghaei & Sharifi, 2022; Seerangurayar et al., 2017a). Additional factors responsible for the reduction in the content of active constituents include microsphere formation during lyophilization caused by the dispersal of bioactive principles inside the configuration of encapsulating wall materials and the development of micropores in the microspheres described above, frequent with sublimation process during lyophilization (Ramírez et al., 2015). This result coincides with previous studies in which higher carrier concentration provoked decreases in the TPC content of spray‐dried amla juice and tea extract powders (Mishra et al., 2015; Şahin Nadeem et al., 2011). Similarly, the phenolic content in freeze‐dried polyherbal formulation using gum Arabic, gelatin, and MD as agents all experienced significant reductions in TPC (Hussain et al., 2018). Moreover, the encapsulation treatment led to lower antioxidant activity due to reduced phenolic and monomeric anthocyanin levels with the 10% MD samples (EC_50_ = 56 ppm) retaining the highest activity compared to the control (EC_50_ = 48 ppm; Figure 3b).

**FIGURE 4:**
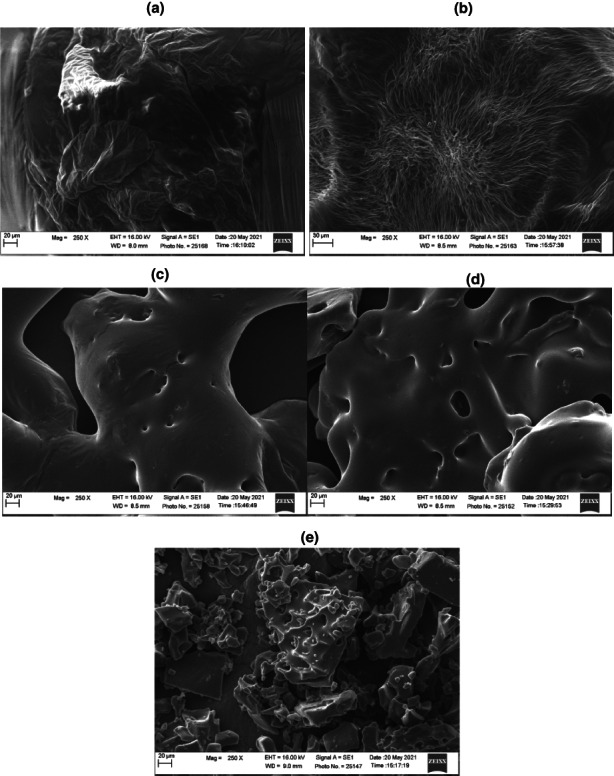
SEM micrograph of foam‐mat freeze‐dried FBGR powder produced with WPI 7.5% and various MD concentration: 0% (a) 10% (b) 20% (c), 30% (d), and 40% (e)

In contrast (Figure 3a), the retention of total monomeric anthocyanins and phenolic compounds in the formulations was significantly improved with higher MD concentration. Retention of phenolic compounds increased from 67% to 76% with the highest retention noted in powders containing 40% MD. Similarly, monomeric anthocyanin retention in the formulations increased from 78% to 84% as MD content increased from 0% to 40%. In freeze‐dried blackberry powder, Franceschinis et al. (2014) reported that after the addition of MD, the retention of anthocyanins and phenolics were 75% and 73%, respectively. In a similar fashion, freeze‐dried Andean blueberry powders retained significantly more monomeric anthocyanins when MD concentration increased from 20% to 30% (Estupiñan‐Amaya et al., 2020). De Souza et al. (2015) also observed high anthocyanin retention (88.36% to 97.35%) in powdered pigment from the Bordo grape when a higher MD concentration was used as a carrier agent (De Souza et al., 2015). For encapsulating bioactive compounds, MD has been shown to offer the best retention of phenolic compounds and flavonoids within the matrix, as well as the best functional properties, particularly when freeze‐drying is used (Ballesteros et al., 2017).

## 4 CONCLUSION

Fermented black glutinous rice (FBGR) powder was produced with the addition of whey protein isolate (WPI) 7.5% (w/w) and maltodextrin (MD) at four concentrations (10%, 20%, 30%, and 40 w/w) by foam‐mat freeze‐drying method. The addition of WPI and MD to FBGR juice had a significant effect on the physicochemical properties resulting in a product with excellent powder properties. FBGR powders with carrier agents had lower moisture content and water activity in addition to improved powder recovery. The foam‐mat freeze‐drying method also produced FBGR powders with high bulk density, high solubility (>70%), and short rehydration time. The use of a carrier agent proved to be an effective technique for encapsulating bioactive components in FBGR, as it retained both phenolic compounds and anthocyanins to support its use as a functional food ingredient. In future research, shelf stability on FBGR powder should be investigated for its potential use in food systems as well as its bioactivity.

## CONFLICT OF INTEREST

The authors declare no conflict of interest.

## Data Availability

The datasets generated during and/or analyzed during the current study are not publicly available due to sponsorship agreement, but can be requested from the corresponding author.
